# Diplopia, Convergent Strabismus, and Eye Abduction Palsy in a 12-Year-Old Boy with Autoimmune Thyroiditis

**DOI:** 10.1155/2016/5823137

**Published:** 2016-06-09

**Authors:** Pedro Marques, Sandra Jacinto, Maria do Carmo Pinto, Catarina Limbert, Lurdes Lopes

**Affiliations:** ^1^Department of Endocrinology, Instituto Português de Oncologia de Lisboa, Lisbon, Portugal; ^2^Department of Pediatric Neurology, Hospital Dona Estefânia, Lisbon, Portugal; ^3^Adolescent Unit, Department of Pediatrics, Hospital Dona Estefânia, Lisbon, Portugal; ^4^Pediatric Endocrinology Unit, Department of Pediatrics, Hospital Dona Estefânia, Lisbon, Portugal

## Abstract

Pseudotumor cerebri (PTC) is defined by clinical criteria of increased intracranial pressure, elevated intracranial pressure with normal cerebrospinal fluid (CSF) composition, and exclusion of other causes such tumors, vascular abnormalities, or infections. The association of PTC with levothyroxine (LT4) has been reported. A 12-year-old boy has been followed up for autoimmune thyroiditis under LT4. Family history was irrelevant for endocrine or autoimmune diseases. A TSH level of 4.43 *μ*UI/mL (0.39–3.10) motivated a LT4 adjustment from 75 to 88 *μ*g/day. Five weeks later, he developed horizontal diplopia, convergent strabismus with left eye abduction palsy, and papilledema. Laboratorial evaluation revealed elevated free thyroxine level (1.05 ng/dL [0.65–1.01]) and low TSH, without other alterations. Lumbar puncture was performed and CSF opening pressure was 24 cm H_2_O with normal composition. Blood and CSF cultures were sterile. Brain MRI was normal. LT4 was temporarily discontinued and progressive improvement was observed, with a normal fundoscopy at day 10 and reversion of diplopia one month later. LT4 was restarted at lower dose and gradually titrated. The boy is currently asymptomatic. This case discloses the potential role of LT4 in inducing PTC. Despite its rarity and unclear association, PTC must be seen as a potential complication of LT4, after excluding all other intracranial hypertension causes.

## 1. Introduction

Pseudotumor cerebri (PTC) is defined by clinical criteria that include symptoms and signs of increased intracranial pressure such headache, papilledema, nausea, emesis, retrobulbar pain, diplopia, and amaurosis, associated with elevated intracranial pressure with normal cerebrospinal fluid (CSF) composition after exclusion of other causes of intracranial hypertension (IH), namely, tumors, vascular abnormalities, or infections [1–3].

*Criteria for the Diagnosis of Pseudotumor Cerebri*

 Signs and symptoms of elevated intracranial pressure.
 No localizing neurological findings.
 Normal cranioencephalic magnetic resonance/computed tomography, excluding tumors or venous sinus thrombosis.
 Increased intracranial pressure over 20 cm H_2_ O (in nonobese patients) or 25 cm H_2_ O (in obese patients) and normal cerebrospinal fluid composition.
 No other identified causes of intracranial hypertension.

The etiology of PTC can be difficult to establish. Several factors may contribute to PTC [4–6].

*Predisposing Conditions for Pseudotumor Cerebri*

 
*Medications*. Amiodarone, corticosteroid (use and withdrawal), cytarabine, desmopressin nasal spray, fluoroquinolones, indomethacin, isotretinoin,* levothyroxine*, lithium, nalidixic acid, nitrofurantoin, oral contraceptives, praziquantel, recombinant growth hormone, and tetracyclines.
 Hypervitaminosis A.
 Immunizations.
 Toxins.
 Pregnancy.
 Cranial trauma.
 Refeeding and weight gain in nutritionally deprived patients.
 
*Systemic Disorders*. Anemia, Behcet's syndrome, coagulation disorders, sleep apnea, systemic lupus erythematosus, and uremia.
 
*Metabolic Disorders.* Addison's disease, hypocalcemia in vitamin D deficiency, obesity, panhypopituitarism, parathyroid dysfunctions (hypo- or hyperparathyroidism), polycystic ovary syndrome, and* thyroid diseases* (hypo- or hyperthyroidism disorders).

The symptoms of PTC in pediatric setting are typically less severe than in adults, but neurological and visual complications may occur; thus, preservation of vision is the main concern and should be promptly addressed [7].

Autoimmune thyroiditis (AIT), the most common thyroid disorder in children and adolescents, is frequently detected in euthyroid phase or just when thyrotropin (TSH) is slightly elevated with no manifestations of thyroid dysfunction [8, 9]. Levothyroxine (LT4) is used to treat hypothyroidism. Considering the consequences of hypothyroidism and hyperthyroidism, the titration of LT4 must be performed at the minimum dosage achieving the desired levels of serum free thyroxine (FT4) and TSH. The main complications associated with LT4 therapy arise from overdose (palpitations, tremor, agitation, hyperactivity, weight loss, or fever) or allergic reactions to the synthetic formulation [10]. However, extremely rare side effects of LT4, reported in the literature such as LT4-induced PTC, may also occur [7, 11].

We present a rare case of PTC associated with LT4 replacement therapy in a pediatric patient with a long standing AIT. This complication occurred shortly after an increase in the dose of LT4 and rapidly reverted after its transient withdrawal.

## 2. Case Presentation

A 12-year-old boy has been followed for AIT with overt hypothyroidism treated with LT4 since the age of 6. Family history was irrelevant for endocrine or autoimmune diseases. His delivery occurred at 32 weeks of gestation and cardiovascular resuscitation was need. Nevertheless motor and cognitive development were normal. At 6 years of age, a downward crossing tendency of the length centiles was noticed (50th to 3rd centile) suggesting a decrease of growth velocity. The physical examination was normal, except for short stature and dry skin. Laboratorial evaluation was compatible with primary autoimmune hypothyroidism: TSH = 395.59 *μ*UI/mL (0.35–4.94); FT4 < 0.4 ng/dL (0.39–1.71); and antithyroid peroxidase and antithyroglobulin antibodies were positive. Thyroid ultrasound revealed a nonnodular diffuse hypoechoic gland compatible with AIT. The boy was started on LT4 and normal growth was resumed.

In December 2013, a TSH level of 4.43 *μ*UI/mL (0.39–3.10) motivated a LT4 dose adjustment from 75 to 88 *μ*g/day (2.34 *μ*g/kg/day). Five weeks later, the patient was referred to our hospital due to a 2-week horizontal diplopia with progressive worsening and had one episode of vomiting. He denied headaches, visual loss, changes in mental status, fever, or recent infectious intercurrence, as well as any recent trauma. On admission, neurological examination identified convergent strabismus with left eye abduction palsy and homonymous diplopia; at fundoscopy bilateral absence of spontaneous venous pulsation was apparent, as well as an elevation of temporal and superior disc margins, suggesting bilateral papilledema. His blood pressure was normal. Laboratorial evaluation revealed increased levels of FT4 (1.05 ng/dL [0.65–1.01]) and low TSH level (0.30 *μ*UI/mL [0.39–3.10]). Complete hemogram, coagulation, liver and renal function, phosphocalcium metabolism, reactive-C protein, 24-hour-urinary free cortisol, angiotensin converting-enzyme, and complement system were within normal range. The titration of the antinuclear, anti-dsDNA, anti-Smith, anticardiolipin IgG/IgM, and anti-beta-2-glycoprotein-1 IgG/IgM serum antibodies was negative. A lumbar puncture was performed and the CSF opening pressure was 24 cm H_2_O; the CSF was limpid, with no cells and normal glucose and proteins concentrations. Blood and CSF cultures were both sterile. Brain magnetic resonance excluded tumors or vascular abnormalities. The patient was started on acetazolamide (375 mg/day), LT4 was discontinued, and a progressive improvement was observed. A normal fundoscopy was found at day 10 and full reversion of diplopia was achieved one month later. Optical coherence tomography showed borderline peripapillary retinal nerve fiber layer thickness suggesting recently resolved papilledema. At that point, the boy was restarted on LT4 at a lower dose with gradual increasing to 75 *μ*g/daily (1.81 *μ*g/kg/day). Under this dose, he is currently asymptomatic with TSH and FT4 within the reference range.

## 3. Discussion

PTC is a rare pediatric condition which implies the exclusion of other entities responsible for IH such as brain tumors, vascular abnormalities, or infectious or autoimmune diseases. Several conditions may be associated with PTC (as mentioned in “Predisposing conditions for pseudotumor cerebri”). A number of medications and other substances have been linked to PTC. A true association implies temporal proximity between the drug initiation/titration, evidence of the association in a substantial number of patients, resolution with drug cessation/titration, and lack of recurrence off medication. Strong evidence associating PTC with tetracyclines, isotretinoin, corticosteroids, or growth hormone is available [3, 12–14]. More limited data to support other drug-induced PTC associations is available. Therefore, clinicians who identify possible associations should report them. A comprehensive review of the literature identified 11 reports outlining the association of LT4 and PTC [7, 15–24].

Although the pathogenesis of PTC is unknown, the elevated cerebral blood volume, the osmotic or vasogenic cerebral edema, the increased CFS outflow resistance at arachnoid villi, and/or the high cerebral venous pressure has been postulated as possible IH mechanisms [1]. The increased resistance to CSF absorption is thought to be caused by an insufficient driving pressure gradient from the subarachnoidal space to the venous system and it is being claimed as the main mechanism [1, 2, 25]. Thyroxine has been proposed as major regulator of sodium transport, raising the cerebral blood flow and the venous pressure and consequently altering the CSF dynamics, which may justify the association between LT4 and PTC. LT4 also increases the CSF production due to its direct effect on the choroid plexus [25, 26]. Moreover, some cases of PTC due to hyperthyroidism were reported [26–29], strengthening the possible association between thyroxine excess and PTC. Furthermore, there are some case reports identifying PTC as a possible side effect from LT4 therapy.

We strongly believe that the occurrence of PTC in our patient was associated with LT4. The majority of LT4-induced PTC cases occur shortly after initiation/upward titration of the LT4 dose [7, 22]. In our patient, a chronological relationship between dose titration and PTC was identified. Secondly, there was a laboratorial documentation of LT4 excess. Thirdly, a detailed anamnesis and clinical evaluation, together with laboratorial and imagiological investigation, ruled out entities like brain tumors, abscesses, venous thrombosis, sinus stenosis, intracranial hemorrhages, arteriovenous malformations, malignant hypertension, infections, autoimmune disorders, anemia, trauma, immunizations, or toxins. Moreover, the resolution of the papilledema within 10 days after LT4 withdrawal, considerably faster than the average 4.7-month resolution time [5], strengths the presumable association between LT4 and PTC. In addition, the resolution of PTC with no need for specific therapies, such as antibiotics, antivirals, corticosteroids, or immunosuppressive agents, excluded other etiologies (bacterial infections, viral infections, and autoimmune diseases). Finally, the lack of recurrence after a significant LT4 dose reduction corroborates this hypothesis.

Currently, there is no consensus on how to approach LT4-induced PTC. Considering the spontaneous resolution capacity of PTC and that in most cases treatment for IH was not required, some authors propose not to change the thyroid replacement regimen [24]. Others defend that a reduction in LT4 dose may be beneficial to the resolution of PTC [16, 19, 22]. In our case, temporary interruption of LT4 allowed the patient to recover, although the use of acetazolamide, justified because of the vision-threatening condition, may have accelerated the recovery. Following our experience, when LT4-induced PTC is suspected, it seems reasonable to shortly stop or reduce the dose of LT4, in order to attenuate or remove the physiological effects of thyroxine in CSF dynamics. If prompt resolution of PTC is required, determined by the severity of neuroophthalmic complications, acetazolamide can be used due to its ability to reduce CSF production. In severe cases furosemide, corticosteroids, immunosuppressive agents, and topiramate may be necessary; surgical interventions must be reserved for cases refractory to medical therapy [1, 2, 4]. LT4 reinitiation in these patients must be cautious and progressive, aiming to achieve the lowest dose and avoiding the development of a similar condition.

To summarize, LT4 replacement therapy is globally safe. However, the clinicians must be aware that PTC may arise in patients under LT4. Despite its extreme rarity, LT4-induced PTC must be seen as a potential complication of LT4 replacement therapy, after excluding all the other IH possible causes, which can be vital to ensure prompt diagnosis and adequate management. Restoration of euthyroidal state should be seen as mainstay approach in case of LT4-induced PTC.
